# *In Vitro* CRISPR/Cas9-Directed Gene Editing to Model LRRK2 G2019S Parkinson’s Disease in Common Marmosets

**DOI:** 10.1038/s41598-020-60273-2

**Published:** 2020-02-26

**Authors:** Scott C. Vermilyea, Alexander Babinski, Nina Tran, Samantha To, Scott Guthrie, Jillian H. Kluss, Jenna Kropp Schmidt, Gregory J. Wiepz, Michael G. Meyer, Megan E. Murphy, Mark R. Cookson, Marina E. Emborg, Thaddeus G. Golos

**Affiliations:** 1 Neuroscience Training Program, University of Wisconsin-Madison, Madison, WI USA; 2 Wisconsin National Primate Research Center, University of Wisconsin-Madison, Madison, WI USA; 3 Cell Biology and Gene Expression Section, Laboratory of Neurogenetics, National Institute on Aging, National Institutes of Health, Bethesda, MD USA; 4 Department of Medical Physics, University of Wisconsin-Madison, Madison, WI USA; 5 Departments of Comparative Biosciences and Obstetrics and Gynecology, University of Wisconsin-Madison, Madison, WI USA; 6 Present Address: Department of Neuroscience, University of Minnesota-Twin Cities, Minneapolis, MN USA

**Keywords:** Cellular neuroscience, Parkinson's disease

## Abstract

Leucine-rich repeat kinase 2 (LRRK2) G2019S is a relatively common mutation, associated with 1–3% of Parkinson’s disease (PD) cases worldwide. G2019S is hypothesized to increase LRRK2 kinase activity. Dopaminergic neurons derived from induced pluripotent stem cells of PD patients carrying LRRK2 G2019S are reported to have several phenotypes compared to wild type controls, including increased activated caspase-3 and reactive oxygen species (ROS), autophagy dysfunction, and simplification of neurites. The common marmoset is envisioned as a candidate nonhuman primate species for comprehensive modeling of genetic mutations. Here, we report our successful use of CRISPR/Cas9 with repair template-mediated homology directed repair to introduce the LRRK2 G2019S mutation, as well as a truncation of the LRRK2 kinase domain, into marmoset embryonic and induced pluripotent stem cells. We found that, similar to humans, marmoset LRRK2 G2019S resulted in elevated kinase activity. Phenotypic evaluation after dopaminergic differentiation demonstrated LRRK2 G2019S-mediated increased intracellular ROS, decreased neuronal viability, and reduced neurite complexity. Importantly, these phenotypes were not observed in clones with LRRK2 truncation. These results demonstrate the feasibility of inducing monogenic mutations in common marmosets and support the use of this species for generating a novel genetic-based model of PD that expresses physiological levels of LRRK2 G2019S.

## Introduction

Leucine-rich repeat kinase 2 (LRRK2) G2019S is a relatively common cause of Parkinson's disease (PD)^1^. Located in the kinase domain of LRRK2, G2019S is hypothesized to slow transition of the kinase from active to inactive forms, thus effectively increasing Vmax of the enzyme by ~2 fold. This increased kinase activity has been identified as the basis of many PD-associated dysregulated intracellular mechanisms^2–7^. Relative to other common PD-associated gene mutations (e.g. SNCA, Parkin, PINK1, GBA), LRRK2 G2019S has a relatively high penetrance^1,8,9^. The prevalence, penetrance, and sequence conservation of this mutation makes it an excellent target for genetic modeling of PD in a nonhuman species.

Because of its genetic similarity to the human genome and shorter lifespan compared to rhesus monkeys^10^, the common marmoset (*Callithrix jacchus*) has emerged as a candidate nonhuman primate species for modeling age-related disorders, including PD. Genetic approaches for modeling PD (or other diseases) in monkeys has been achieved by introducing the mutant gene using viral vector technologies, via direct intracerebral delivery or microinjection delivery in the oocyte^11,12^. In both cases, the mutant protein is over-expressed at supraphysiological levels. In addition, intracerebral delivery requires brain surgery, and its effects are limited to the target region, which contradicts the current understanding of neurodegeneration as a multisystem disease.

Methods for precise genomic editing have improved significantly with the recent ability to harness a part of the bacterial innate immune system, known as clustered regularly interspaced short palindromic repeats (CRISPR/Cas9), to easily target specific gene sequences and induce double-stranded DNA breaks. CRISPR/Cas9 relies on endogenous mechanisms of non-homologous end joining for activating DNA repair, which is prone to errors upon blunt end repair^13^. As a research alternative, single nucleotides can be edited with the addition of repair templates that act as homology arms^14^. Most recently CRISPR/Cas9 was used to manipulate the common marmoset genome for genetic modeling purposes^15^.

Here we present a CRISPR/Cas9 gene editing method to introduce the PD LRRK2 G2019S monogenic mutation in marmoset embryonic (Cj-ESC) and induced pluripotent (Cj-iPSCs) stem cells. An editing cassette targeting LRRK2 exon 41 was used in combination with a 141-nucleotide (nt) single-stranded oligodeoxynucleotide (ssODN) to promote homology-directed repair (HDR) of the Cas9-mediated double-stranded DNA break. We found that similar to humans, the common marmoset LRRK2 G2019S mutation resulted in elevated kinase activity. Phenotypic evaluation of dopaminergic neurons derived from the mutant marmoset stem cell lines showed LRRK2 G2019S-mediated changes in cell homeostasis and neurite branching. These methods will aid in the development of novel genetic-based models of PD LRRK2 G2019S, as well as new gene targets in marmosets and other nonhuman primate species.

## Results

### Targeting of the common marmoset LRRK2 gene using CRISPR/Cas9

**Figure 1:**
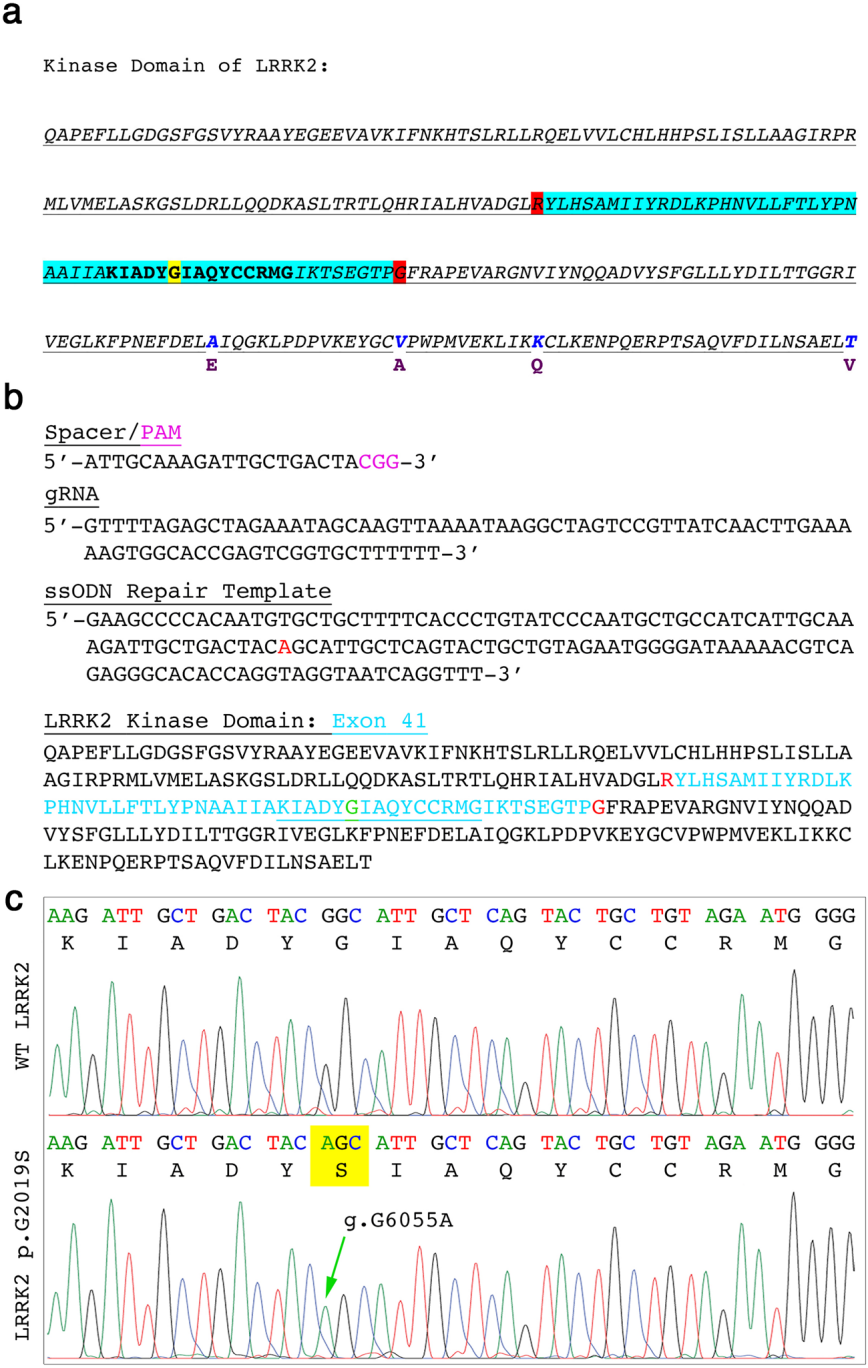
CRISPR/Cas9 targeting of marmoset LRRK2 exon 41. (**a**) Marmoset LRRK2 kinase domain showing exon 41 (aqua), targeted glycine (yellow), exon junctions (red) and amino acid substitutions compared to the human LRRK2 kinase amino acid sequence (human amino acids in purple below the marmoset residues). (**b**) The genetic spacer sequence design illustrated with the adjacent PAM sequence cloned in to express continuous with the gRNA hairpin sequence. A 141-nucleotide repair template with the g.G6055A-targeted edit (red) was used to direct HDR. The underlined portion including the targeted glycine (green) is further evaluated below. (**c**) Representative wild type (WT) marmoset LRRK2 sequence (upper panel) showing codons and translated amino acid residues and an edited marmoset cell line showing the homozygous g.G6055A mutation that translates to p.G2019S (lower panel).

To evaluate whether it was feasible to introduce the PD LRRK2 G2019S mutation in the marmoset genome, we first compared marmoset and human LRRK2 protein sequences. We found high homology across the protein sequence, including the well-conserved kinase domain (Fig. 1a). A guide RNA (gRNA; ATTGCAAAGATTGCTGACTA) was then designed so that the 20-nucleotide variable domain was homologous to the g.G6055 site, and also compatible with a protospacer adjacent motif (PAM) (Fig. 1b). Proper HDR of the g.G6055 further prevents subsequent binding of the gRNA:Cas9 complex as it disrupts the PAM motif. The synthesized 20-nucleotide spacer was cloned into the lentiCRISPR-V1-gRNA/puromycin selection expression cassette with dual gRNA and Cas9 expression from a single vector system. The addition of a 141-nucleotide single-stranded oligodeoxynucleotide (ssODN) led to successful g.G6055A repair (Fig. 1c). The top eight loci that were predicted to be potential off-target sites for the gRNA were individually sequenced in two homozygous LRRK2 G2019S clones derived from both Cj-ESCs and Cj-iPSCs (Supplemental Table 1). No mutations were observed at any of the eight loci within any of the four mutant clones. In addition, none of the analyzed loci were located within annotated genes in the marmoset genome. Cross-sequence analysis to the more highly annotated human genome predicted one of the sites on chromosome 2 to be associated with a transcript variant of methyltransferase like 22 (*METTL22*), and solute carrier family 25 member 53 (*SLC25A53*), which could be a homologous reading frame in the marmoset.

### Midbrain dopaminergic differentiation of LRRK2 G2019S Cj-ESCs and Cj-iPSCs

**Figure 2:**
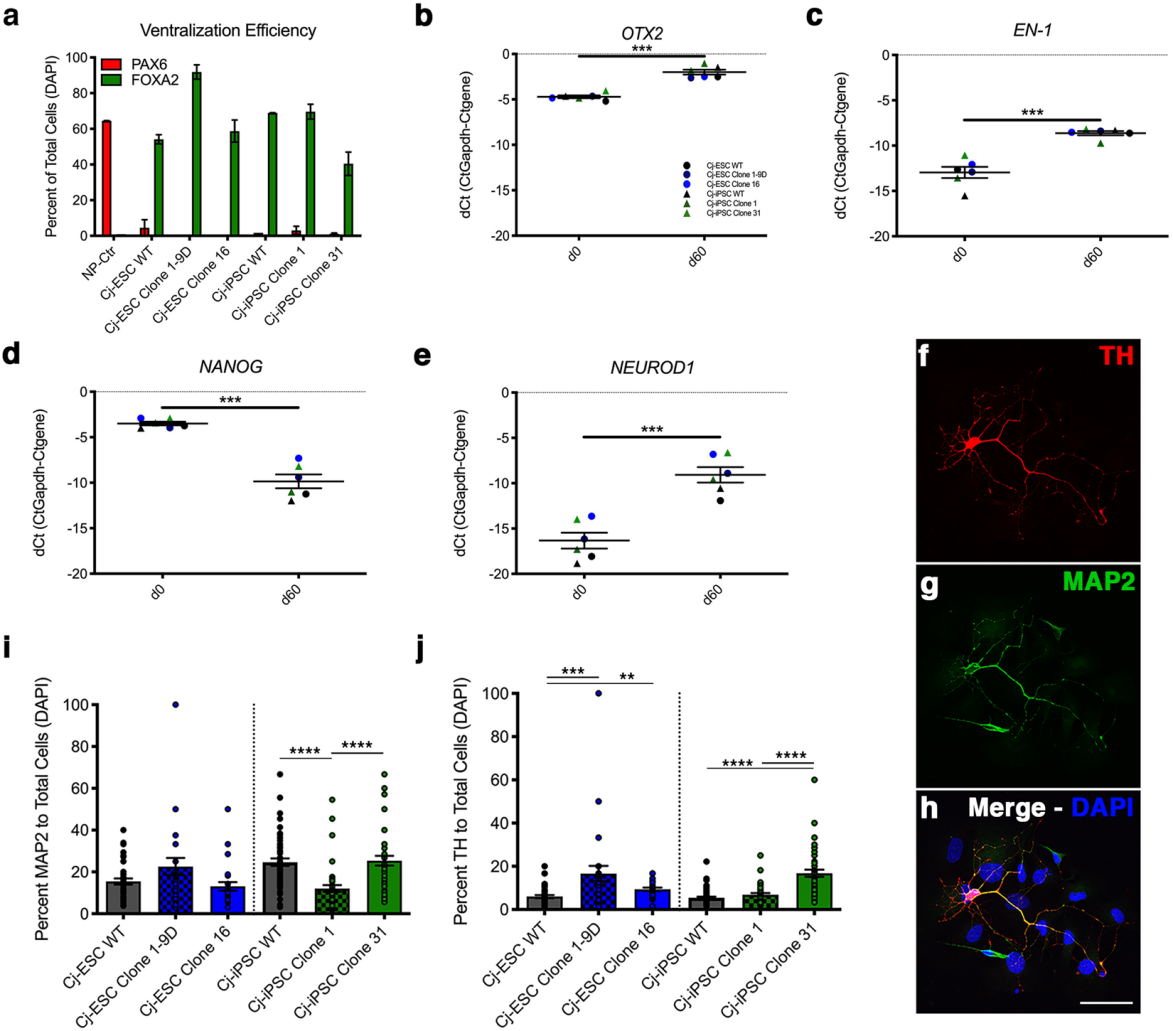
Patterning to floorplate-derived midbrain dopaminergic neurons. (**a**) Quantification of the dorsal PAX6 marker and ventral floorplate marker FOXA2 in Cj-ESC and Cj-iPSC LRRK2 G2019S cell lines and their respective parental wild type (WT) lines. One cell line was differentiated without patterning as a control for PAX6 and FOXA2 staining (NP-Ctr). Data was collected on day 18 (**b**,**c**) qRT-PCR for the midbrain markers *OTX2* and *EN1* comparing d0 and d60 of all six lines combined. (**d,e**) qRT-PCR for the pluripotent gene *NANOG* and neuronal differentiation gene *NEUROD1* in all six lines at d0 and d60 of dopaminergic differentiation. (**f–h)** Example of TH+/MAP2+ and TH−/MAP2+ neurons. (**i,j**) Quantification of MAP2 and TH positive stained neurons to the total number of DAPI nuclei within each captured field. For differentiation efficiency analysis, each data point represents a separate captured field. Panels b-e: Cj-ESCs (circles: wt – black; clone 1–9D, dark blue; clone 16, blue) and Cj-iPSCs (triangles: wt – black; clone 1, dark green; clone 31, green) Scale bar: 25 μm. (Students’ t-test was performed to compare timepoints; Kruskal-Wallis test with Dunn’s multiple comparisons test was performed to compare across clones; p < 0.01^**^; p < 0.001^***^; p < 0.0001^****^).

Neurodegeneration of midbrain dopaminergic neurons is a pathological hallmark of PD^16^. Thus, we assessed the impact of the mutation on the dopaminergic differentiation potential of genomic edited cell lines. Parental wild type and two homozygous G2019S mutant clones were differentiated in parallel from both Cj-ESCs and Cj-iPSCs using our previously validated methods^17^. Confluency and colony expansion were kept consistent across all six lines during the initial neural induction and patterning. During the differentiation (day 18), ventralization patterning efficiency was quantified from each of the six lines to confirm proper conversion from PAX6+/FOXA2− to PAX6−/FOXA2+ cells (Fig. 2a). Midbrain posteriorization was confirmed by qRT-PCR identification of significant increase of *OTX2* and *EN-1* expression after 60 days of dopaminergic differentiation (Fig. 2b,c). In addition, qRT-PCR of pluripotency gene *NANOG* and neural differentiation gene *NEUROD1* showed significant decrease and increase of expression, respectively (Fig. 2d,e). Quantification of microtubule associated protein 2 positive (MAP2+) neurons and tyrosine hydroxylase positive (TH+) dopaminergic neurons showed variability among cell lines (Fig. 2f–j). For Cj-ESC-derived lines, differences in the number of MAP2+ neurons were not detected, although significantly fewer TH+ neurons were derived from wildtype compared to G2019S clones. With respect to Cj-iPSC-derived neurons, significantly fewer MAP2+ neurons were produced by Clone 1 compared to wildtype and Clone 31, while significantly less TH+ neurons were produced by wildtype and Clone 1 compared to Clone 31.

### Common marmoset LRRK2 G2019S kinase activity after dopaminergic differentiation

**Figure 3:**
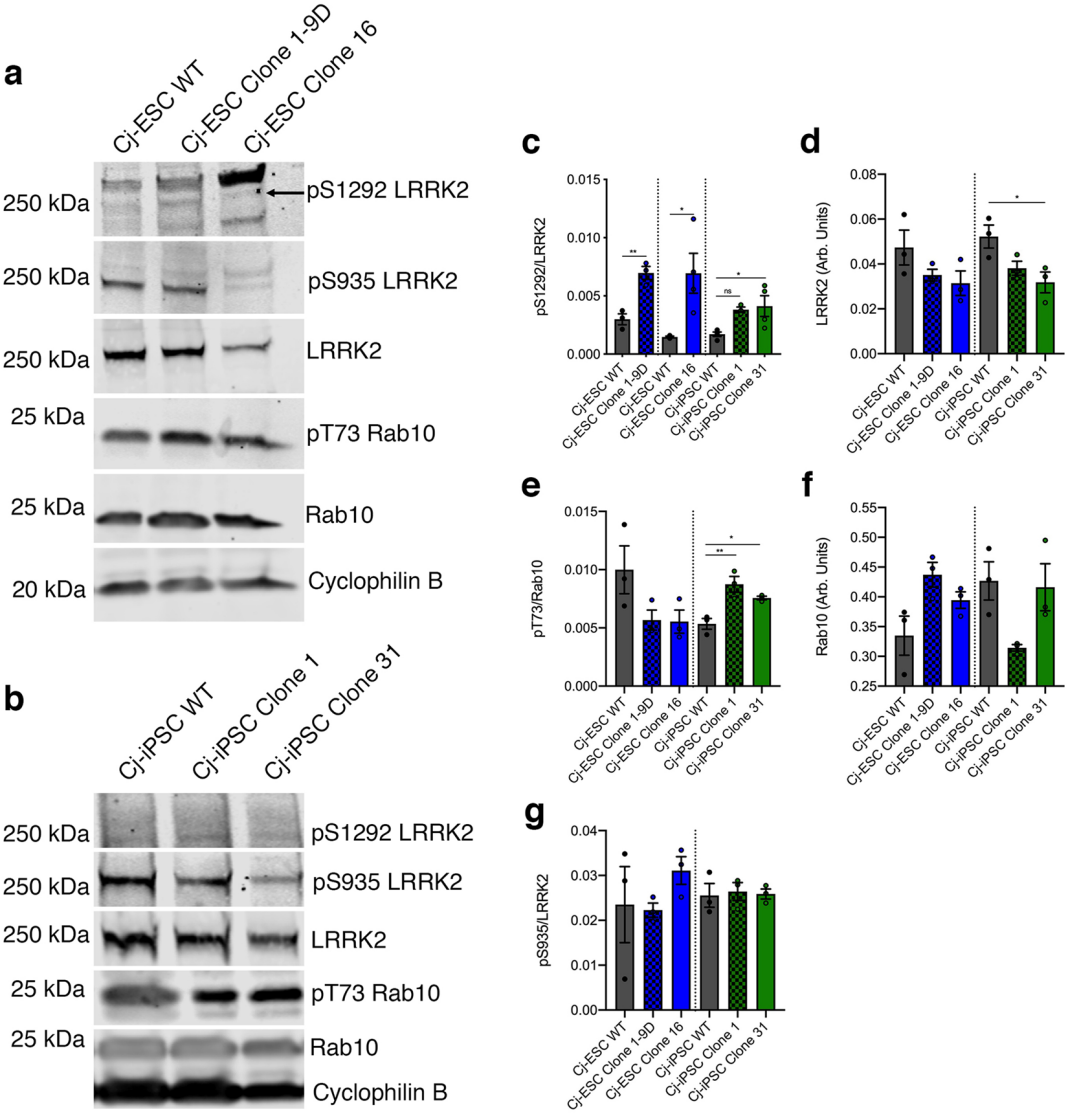
Marmoset LRRK2 kinase assay. (**a**) Representative Western Blot for pS1292 LRRK2 autophosphorylation, pS935 LRRK2, LRRK2, pT73 Rab10, Rab10, and cyclophilin B for Cj-ESC wild type (WT), Cj-ESC Clone 1–9D, and Cj-ESC Clone 16, and (**b**) Cj-iPSC WT, Cj-iPSC Clone 1, and Cj-iPSC Clone 31. (**c**) Relative quantification of pS1292/LRRK2 shows significantly increased pS1292 autophosphorylation in three G2019S clones compared to their respective wild type (WT) line. (**d**) LRRK2 protein expression levels (normalized to cyclophilin B) were consistent except for a significant decrease in Cj-iPSC Clone 31. (**e**) Relative quantification of pT73/Rab10 shows variability between Cj-ESC and Cj-iPSC lines but with significant increases in both Cj-iPSC G2019S clones. (**f**) Rab10 expression (normalized to cyclophilin B) was variable between all lines but without any significant difference. (**g**) There was no difference among lines for the constitutively phosphorylated pS935 LRRK2; n = 3–4 separately differentiated and collected samples per line. Note: artifact observed at the level of pS1292 detection was not quantified. (One-way ANOVA with Tukey’s multiple comparison was used to compare among Cj-ESC or Cj-iPSC lines. Student’s t-test was used for pS1292 in Cj-ESCs as the data for each G2019S clone was collected independently with respective WT controls; p < 0.05^*^; p < 0.01^**^).

The G2019S mutation in human iPSC-derived dopaminergic neurons is known to increase kinase activity, which has been linked to pathways of neuronal dysfunction^4,7^. After Cj-ESC wild type, Clone 1–9D, and Clone 16, as well as Cj-iPSC wild type, Clone 1, and Clone 31 were differentiated towards a midbrain dopaminergic phenotype, cell lysates were analyzed for markers of LRRK2 kinase activity^18^. Phosphorylation of serine 1292 (pS1292) and Rab10 were used as a measure of kinase activity (Fig. 3a,b). The level of pS1292 was significantly increased (p < 0.05) in all LRRK2 G2019S lines, except for a non-significant difference in Cj-iPSC clone 1, relative to their parental wild type lines (Fig. 3c). Interestingly, all mutant lines showed a decrease in overall LRRK2 protein expression with Cj-iPSC Clone 31 being significantly reduced (Fig. 3d). When analyzing pT73 Rab10, both Cj-iPSC clones 1 and 31 had significantly elevated levels of pT73 compared to wild type, while both Cj-ESC clones did not show significant differences (Fig. 3e). Overall Rab10 expression levels were variable and not significantly different between lines (Fig. 3f). In addition, there was no change in pS935 levels among any lines (Fig. 3g).

### Morphology of differentiated dopaminergic marmoset neurons

**Figure 4:**
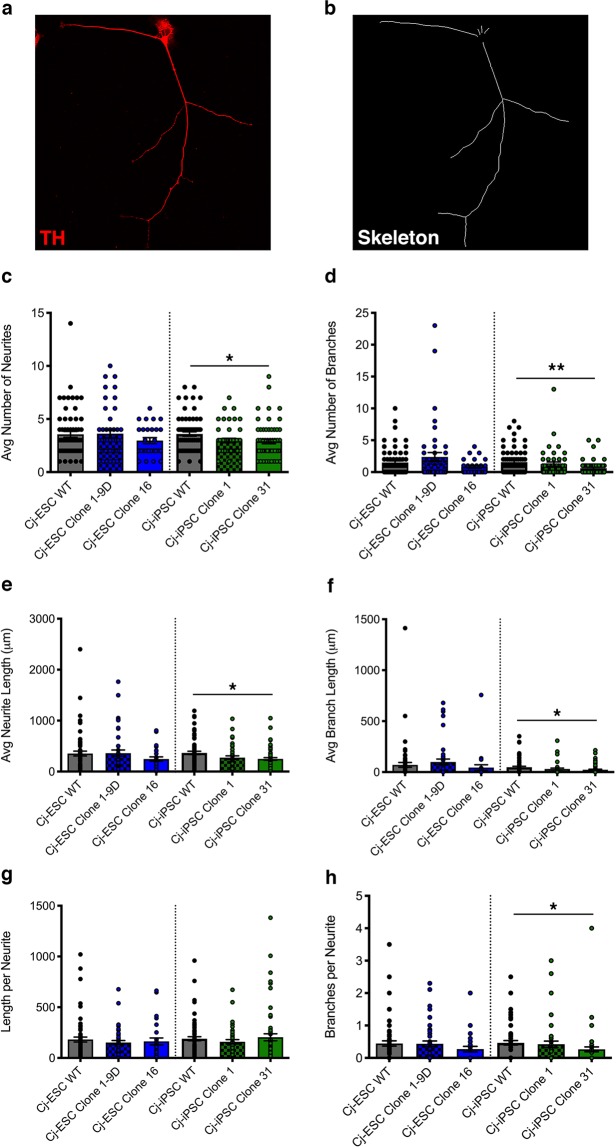
Morphological analysis of wild type (WT) and LRRK2 G2019S TH+ dopaminergic neurons. (**a**) Representative TH+ neuron and (**b**) traced skeleton for morphological quantification. (**c**) Average number of neurites per cell, (**d**) average number of branches per cell, (**e**) average neurite length per cell, (**f**) average branch length per cell, (**g**) length per neurite, and (**h**) branches per neurite were quantified in WT and LRRK2 G2019S Cj-ESC and Cj-iPSC TH+/MAP2+ d67 dopaminergic neurons; n = 25–45 neurons per line. (One-way ANOVA with Tukey’s multiple comparison was used to compare between Cj-ESC lines or between Cj-iPSC lines; p < 0.05^*^, 0.01^**^).

As LRRK2 G2019S iPSC patient-derived dopaminergic neurons show changes in neurite arborization (reviewed by Sison *et al*.^19^), we evaluated the morphology of the differentiated cell lines. Midbrain dopaminergic neurons from both Cj-ESC and Cj-iPSC parental wild type and LRRK2 G2019S clones were fixed and immunostained for the dopaminergic and neuronal markers TH and MAP2 at 67 days after differentiation induction. Neurons that were double-labeled for TH and MAP2 were traced using the ImageJ plugin Simple Neurite Tracer (Fig. 4a–h). Although Cj-ESC Clone 16 and Cj-iPSC Clones 1 and 31 had simplified morphological characteristics compared to their respective parental wild type neurons, Cj-iPSC Clone 31 was the only line to show significantly decreased number of neurites per cell (Fig. 4c), branches per cell (Fig. 4d), neurite length (Fig. 4e), branch length (Fig. 4f), and branches per neurite (Fig. 4h) compared to Cj-iPSC wild type. There was no difference among cell lines for length per neurite.

### Analysis of cell homeostasis after dopaminergic differentiation of wild type and G2019S stem cells

**Figure 5:**
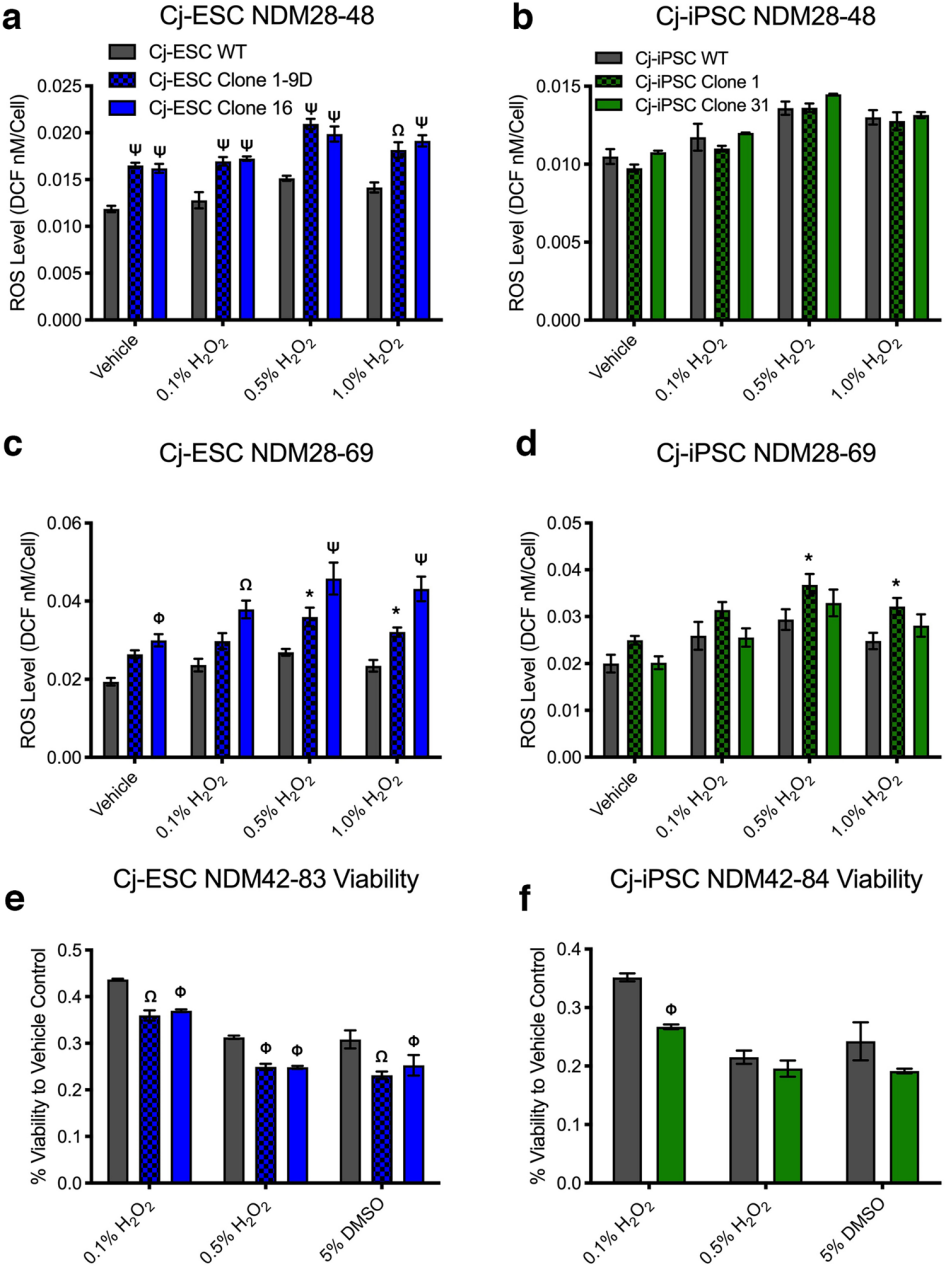
ROS and viability assays of wild type and LRRK2 G2019S neurons. (**a**) Cj-ESCs, and (**b**) Cj-iPSCs assayed for ROS levels after treatment with vehicle, 0.1%, 0.5%, and 1.0% hydrogen peroxide after 20 days of maturation (Graph key represents each column of the figure). (**c**) Cj-ESCs and (**d**) Cj-iPSCs assayed for ROS levels after an additional 20 days of culture. All ROS graphs are shown as a concentration of DCF compound per cell as a readout of intracellular ROS abundance. (**e**) Cj-ESCs and (**f**) Cj-iPSCs assayed for viability after treatment with 0.1%, 0.5% hydrogen peroxide, or 5% dimethylsulfoxide after 40 days of maturation. Viability was normalized to control wells treated with vehicle; n = 3 separate wells per line and condition. (Two-way ANOVA with Bonferroni correction comparing cell lines to condition with significance presented on the graphs only in comparison to wild type: p < 0.05^*^, 0.01^Φ^, 0^.^001^Ω^, 0.0001^Ψ^).

The increased kinase activity of LRRK2 G2019S has been implicated in the dysfunction of several intracellular homeostatic mechanisms^20–22^. As a result, we aimed to evaluate the effects of LRRK2 G2019S on intracellular ROS production, neuronal viability, as well as protein clearance pathway-related transcripts and proteins. The intracellular ROS levels were assayed after 20 and 40 days of final neuronal maturation. After twenty days in neural differentiation media (i.e. NDM day 28–48), intracellular ROS levels were significantly higher in both Cj-ESC LRRK2 G2019S clones compared to wild type in all conditions (vehicle, 0.1%, 0.5%, and 1.5% H_2_O_2_; Fig. 5a) but not when compared with each other. Cj-iPSC derived LRRK2 G2019S dopaminergic neurons did not have higher ROS at the same NDM28-48 time point (Fig. 5b). After an additional twenty days (i.e. NDM28-69), intracellular ROS levels within Cj-ESC Clone 16 were significantly higher than wild type cells in all four conditions while Clone 1–9D ROS levels were only significantly increased after addition of 0.5% and 1.0% H_2_O_2_ (Fig. 5c). In all peroxide conditions for Cj-ESC NDM28-69, Clone 16 ROS levels were also significantly higher than Clone 1–9D. In addition, Cj-iPSC Clone 1 had significantly higher ROS levels after addition of 0.5% and 1.0% H_2_O_2_ compared to wild type cells (Fig. 5d).

A test for neuronal viability in cells plated on day 42 and matured for 40 additional days showed significantly reduced viability in all three LRRK2 G2019S clones after treatment with 0.1% H_2_O_2_, as well as 0.5% H_2_O_2_ and 5% DMSO in Cj-ESC Clone 1–9D and Clone 16, but not Cj-iPSC clone 31 (Fig. 5e,f). Cells differentiated from Cj-iPSC Clone 1 did not survive to this time point.

Expression of autophagy and ER stress related genes were evaluated by qRT-PCR using RNA samples collected on day 60 of dopaminergic differentiation (Supplemental Fig. 2). The autophagy related genes *ATG7* and *LAMP2* did not show any significant difference between wild type or G2019S lines. *LC3A* was significantly increased in Cj-iPSC clone 31, whereas *LC3B* was significantly increased in Cj-ESC Clone 16. *P62* was significantly higher in both Cj-iPSC G2019S clones compared to wild type. *BiP* was increased in Cj-iPSC Clone 1 while the pro-apoptotic gene *CHOP* was increased in both Cj-ESC Clone 16 and Cj-iPSC Clone 31, although there was no significant difference observed in *CASP3* expression. Lastly, there was no difference in the mitochondrial related *DRP1* gene expression.

To evaluate the expression of autophagy-related proteins, protein lysates were collected at d60 of dopaminergic differentiation and Western Blot analysis was performed for LC3 I-II and P62 (Supplemental Fig. 3a,b). P62 was significantly lower in Cj-iPSC G2019S clones compared to their parental wild type, however no difference was observed among Cj-ESCs (Supplemental Fig. 3c). LC3I levels were lower in Cj-iPSC clone 1 and 31, although this was only statistically significant in Clone 31 (Supplemental Fig. 3d), while LC3 II was significantly reduced in both Cj-iPSC G2019S clones, and conversely increased in Cj-ESC Clone 16 (Supplemental Fig. 3e). When comparing the ratio of LC3II/I, only Cj-iPSC Clone 31 demonstrated a reduced ratio (Supplemental Fig. 3f).

### Generation and evaluation of truncated LRRK2

**Figure 6:**
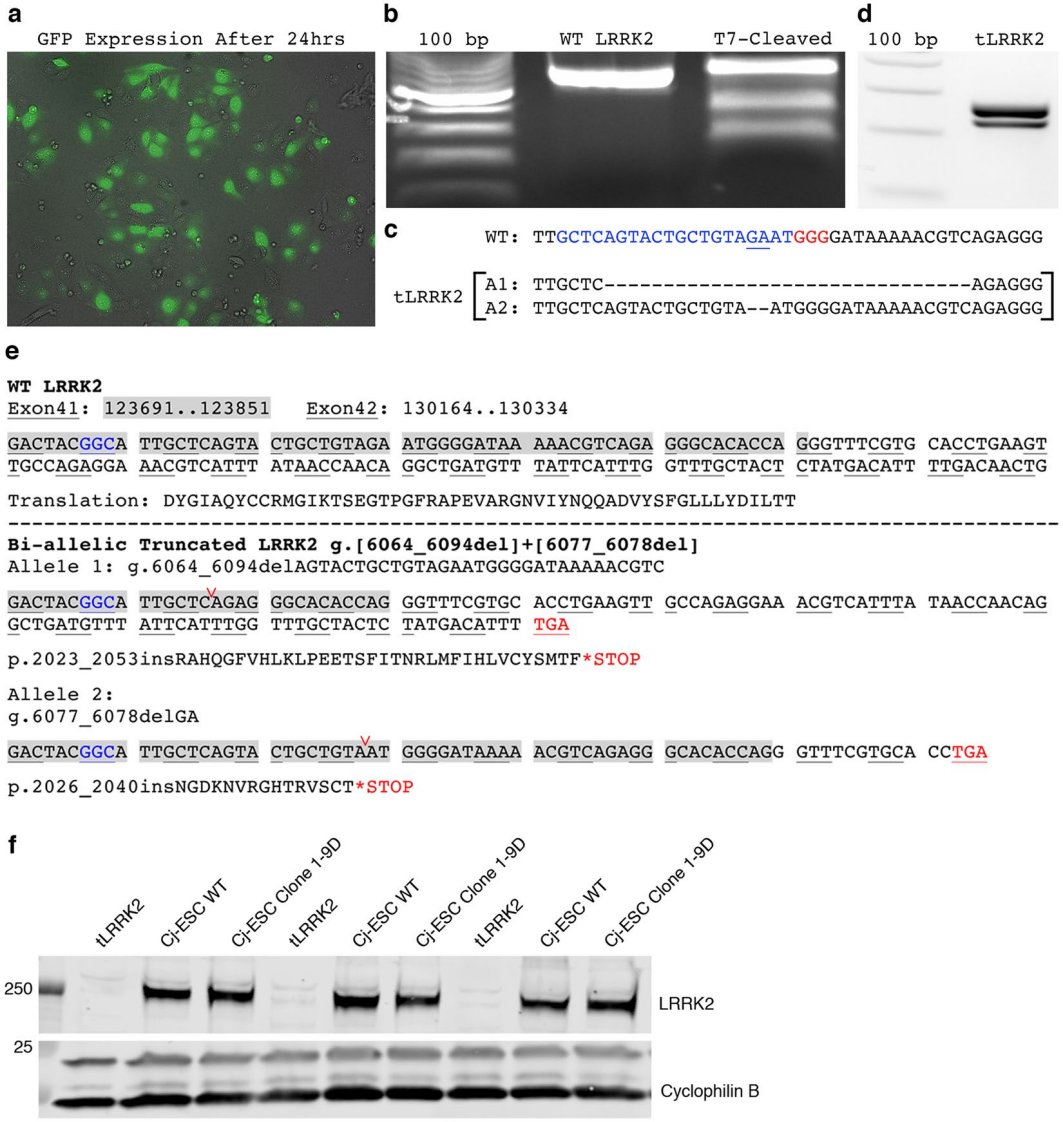
CRISPR/Cas9 truncation of LRRK2 in exon 41. (**a**) GFP expression after electroporation pre-FACS. (**b**) Site-directed cleavage and NHEJ-induced INDEL formation was detected using a T7-endonuclease SURVEYOR assay from GFP sorted cells, which cleaves non-homologous base combinations when wild type amplified pcr products are mixed and annealed with potentially mutant amplicons. (**c**) Next-generation sequencing of the target site in the newly derived cell line presented two distinct allelic deletions (A1, A2; blue denotes gRNA homology target and red denotes PAM in wild type gene sequence). (**d**) RT-PCR of the g.G6055 locus from the single isolated clone shows separation between the new sequence lengths after editing. (**e**) Evaluation of exon 41 (chromosomal bases 123691-123851) and exon 42 (chromosomal bases 130164-130334) in wild type (WT) and the tLRRK2 clone illustrates the generation of frame shift mutations in the gene reading frame sites (g.6064-6094del and g.6077_6078del respectively; deletion sites shown in panel (c) are at the location of the red carat) and ensuing premature stop codons in both alleles (blue text denotes location of p.G2019 codon). (**f**) Western blot for LRRK2 using a C-terminal antibody shows the loss of LRRK2 detection in Cj-ESC tLRRK2 neurons.

The identification of kinase-mediated cellular dysfunction has led to the development of several kinase inhibitors as therapeutic methods for LRRK2 silencing (see review by Zhao and Dzamko^23^). Thus, we evaluated the consequences of deleting a portion of the kinase domain from Cj-ESCs using CRISPR/Cas9-directed NHEJ on cellular homeostatic mechanisms. Cj-ESCs were electroporated with a plasmid expressing Cas9, a gRNA directing to exon 41, and GFP (Fig. 6a) in order to induce truncation of LRRK2 (tLRRK2). Cells that expressed GFP were fluorescently sorted and expanded in culture. The T7 endonuclease SURVEYOR mutation detection assay was first used to demonstrate proper targeting and generation of random insertions and deletions (INDELs) upon double-stranded DNA-breakage repair (Fig. 6b). Next-generation sequencing confirmed an isolated clone with two different frameshift inducing bi-allelic deletions (2 and 31 nucleotide deletions; Fig. 6c) that could be size-distinguished with gDNA PCR gel electrophoresis (Fig. 6d). Further gDNA sequencing analysis of the reading frame revealed this clone contained premature STOP codons as a result of the bi-allelic frame shifts that were predicted to remove the kinase activity of LRRK2 while potentially maintaining the N-terminal domains (Fig. 6e). Loss of C-terminal antibody binding through Western Blot confirmed the prediction of a truncated LRRK2 protein (Fig. 6f).

Off-target sequencing analysis was completed to determine whether binding and INDEL formation occurred as a result of near homologous gRNA annealing (Supplemental Table 2). No mutations were observed in the top eight loci identified as potential off-target binding sites. None of the potential sites were within annotated marmoset genes.

**Figure 7:**
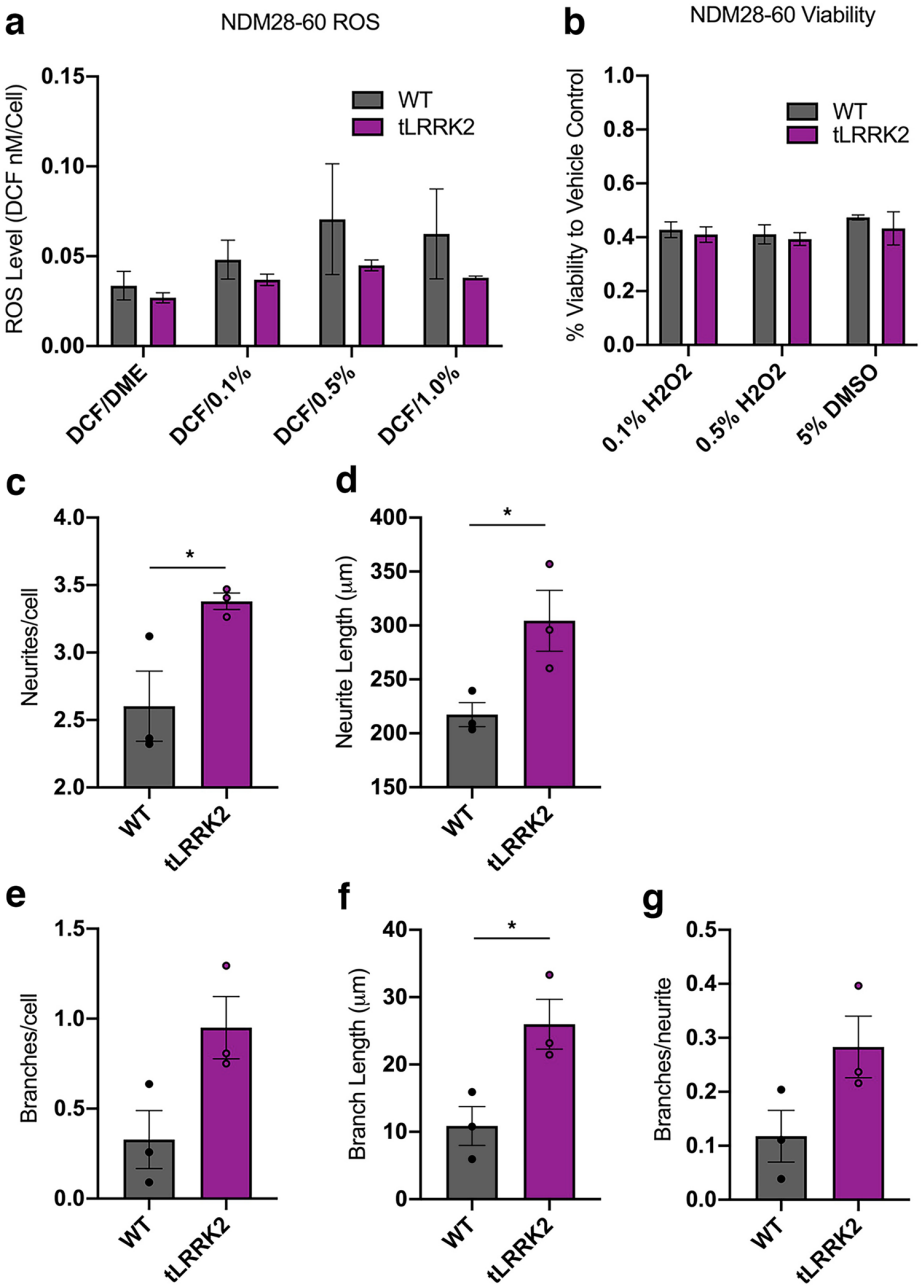
ROS, viability, and morphological assays of wild type and tLRRK2 neurons. (**a**) Cj-ESC wild type (WT) and tLRRK2 dopaminergic neurons assayed for ROS levels after treatment with vehicle (DCF/DME), 0.1%, 0.5%, or 1.0% hydrogen peroxide at 60 days *in vitro*. All ROS graphs are shown as a concentration of DCF compound per cell as a readout of intracellular ROS abundance; n = 3 separate wells per line and condition. (**b**) Cj-ESC wild type and tLRRK2 dopaminergic neurons assayed for viability after treatment of 0.1%, 0.5% hydrogen peroxide, or 5% dimethylsulfoxide at 30 days *in vitro*. Viability was normalized to control wells treated with vehicle; n = 3 separate wells per line and condition. Morphological analysis of Cj-ESC wild type and tLRRK2 neurons in three separate differentiation trials analyzing (**c**) neurites/cell (p = 0.0434), (**d**) neurite length/cell (p = 0.0456), (**e**) branches/cell (p = 0.0582), (**f**) branch length (p = 0.0322), and (**g**) branches/neurite (p = 0.0904); n = 20–30 neurons per line. (Two-way ANOVA with Bonferroni correction was used to test ROS and Viability; Student’s t-test was used for morphological analysis; Asterisk denotes p < 0.05).

Evaluation of intracellular ROS using the intracellular DCF reporter molecule showed no sign of altered ROS abundance at baseline or after burden with increasing hydrogen peroxide concentrations (0.1%, 0.5%. and 1%) (Fig. 7a). Furthermore, tLRRK2 expression did not have any effect on neuronal viability (Fig. 7b). tLRRK2 differentiated midbrain dopaminergic neurons expressed significantly higher EN-1 and TH, with similar OTX2 expression, validating their regional expression (Supplemental Fig. 4). However, no difference in expression of tested autophagy and ER stress related genes were observed between tLRRK2 and parental wild type controls in three separate differentiation experiments (Supplemental Fig. 4).

Differentiation of wild type and tLRRK2 dopaminergic neurons across three individual experimental replicates demonstrates a significant increase in neurite and branch parameters in the tLRRK2 neurons. Significant increases in neurites/cell (p = 0.0434; Fig. 7c), neurite length/cell (p = 0.0456; Fig. 7d), and branch length/cell (p = 0.0322; Fig. 7f) were observed. The number of branches/cell was not significant (p = 0.0582; Fig. 7e), while no difference was observed in branches/neurite (p = 0.0904; Fig. 7g).

## Discussion

The current study is the first demonstration of successful introduction of a disease-related monogenic mutation in marmoset derived stem cells using CRISPR/Cas9. It also demonstrates that LRRK2 G2019S in marmoset dopaminergic neurons leads to increased kinase activity resembling findings in PD patient-derived LRRK2 G2019S cells.

Improved animal models of PD that present progressive and comprehensive multisystem neuropathology are needed. As etiology of sporadic PD is not clear, in the last few years emphasis has been placed in modeling mutation–induced pathology by injections of viral vectors encoding for a gene of interest, either by intracerebral injection in adult animals or by injection into fertilized oocytes to induce transgenesis^11^. A preferred PD-associated gene target has been α-synuclein (SNCA), however, mutations in SNCA are rare and species differences in critical parts of the gene limits the translatability of the findings^12^. For example, a comparison between SNCA gene sequence in humans vs. common marmoset reveals that this new world species has a naturally occurring p.T53. Thus, editing the A53T mutation would be unnecessary, and editing for other SNCA mutations (e.g.: A30P), without humanizing the rest of the SNCA gene, presents a high level of uncertainty for observing relevant pathology. In addition, overexpression of α-synuclein protein, as induced by viral vector gene transfer, is only observed in extremely rare cases of SNCA duplication or triplication^24,25^. Targeting LRRK2, compared to SNCA, presents several advantages for PD modeling, as LRRK2 mutations have a higher incidence and the most affected gene loci, within the kinase region, are conserved between mammalian species. Penetrance of LRRK2 G2019S is not as high as SNCA A53T (~90% by 70 years old) but still is substantial with a recent estimate of 42.5% (95% confidence interval [CI]: 26.3–65.8%) at 80 years of age^1,8,9,26^. Due to the size of the gene (7581 base transcript), LRRK2 is difficult to express using viral-vector gene transfer technologies. In this context, CRISPR/Cas9 emerges as a technology suitable for gene modification to generate animal models of human diseases. Our study establishes the feasibility of inducing point mutations in the common marmoset genome. This validation is the first step towards developing a genomic edited PD monkey model and showcases an approach that could be applied to other genes of interest.

Targeted double-stranded DNA breaks and INDEL formation are relatively easy to achieve, yet point mutations are more difficult. Activating HDR mechanisms with the addition of a repair template has a much lower efficiency and has been the subject of optimization studies^27,28^. Here, we found more efficient repair with a combination of longer repair template, minimal distance between DNA excision and repair location, as well as a selection method for clones electroporated with the plasmid cassettes (data not shown). The use of puromycin-resistance expression plasmids led to improved selection and expansion of edited clones. The gRNA that ultimately performed best for HDR targeted Cas9 to cut the DNA 5 bases in the 5’ direction from the g.G6055 base. Other gRNAs that guided editing 21, 23, and 39 bases away were efficient at targeting, but were unsuccessful at G6055A repair. Similarly, the 141-nucleotide ssODN repair template with 70-nucleotide flanking arms was used after failed experiments using double-stranded templates as well as 90- and 120-nucleotide ssODN templates.

CRISPR/Cas9 gRNA variable domain is designed for site recognition limited to 20-nucleotides, thus other DNA loci with high homology may be subject to off-target annealing and unintended INDEL formation. Evaluation of off-target sites predicted by NCBI BLAST in both our G2019S and tLRRK2 edited clones did not identify INDEL formation. In addition, no sites were located within known annotated genes in the common marmoset, or within genes homologous to the more highly annotated human genome.

The introduction of the LRRK2 G2019S mutation induced phenotypes in the marmoset neurons similar to those observed in PD patient cells^2–7^. Most critically, evaluation of the LRRK2 G2019S mutated cell lines revealed increased LRRK2 kinase activity by identification of elevated LRRK2 S1292 phosphorylation, a known marker of elevated LRRK2 kinase autophosphorylation. The increased phosphorylation was found despite the low (10–20%) efficiency of neuronal differentiation. This result may have been more profound if completed using isolated TH+ neurons, as the limited number of TH+ cells in the cultures likely contributed to the assay variability across lines. In addition, variability in neuronal (i.e. MAP2+) and TH+ dopaminergic neuron generation was found in both Cj-ESC and Cj-iPSC lines. Of note, Cj-ESC wildtype produced significantly less TH+ neurons compared to both LRRK2 G2019S clones while Cj-iPSC wildtype produced significantly less TH+ neurons than Cj-iPSC Clone 31. Although it is unclear as to the factor(s) underlying the differentiation efficiency between these lines, it is plausible that the inconsistent abundance of progenitor/non-neuronal cells across the lines, rather than their ability to differentiate to TH+ neurons is the reason behind the perceived variability. The differences in neuronal and TH+ neuron percent-abundance are likely responsible for much of the cell line variability observed, as evaluation of ROS, viability, RNA, and protein were not specific to individual TH+ neurons. The variability in abundance of neurons compared with non-neuronal or neural progenitor cells in samples collected across lines may, for example, explain Cj-iPSC G2019S cells’ significantly elevated pT73 Rab10 levels, which was not observed in Cj-ESC G2019S cells. Future studies should consider cell culture purification strategies, such as the introduction of mitotic inhibitors or development of a GFP reporter of TH expression to allow isolation of TH+ cell populations to be subsequently assayed.

In PD patient-derived stem cells, increased LRRK2 kinase activity is associated with changes in cellular homeostasis^3^. An assay for intracellular ROS species in marmoset dopaminergic neurons showed robust increased levels of ROS in Cj-ESC LRRK2 G2019S lines compared to their parental wild type line, although this was not as clear in the Cj-iPSCs. These differences may be related to the reprogramming event, or parental cell line variability. Older mitochondrial age of adult-derived iPSC lines compared to ESCs may have contributed to the differences in cellular homeostasis^29–31^, although the variability is apparent across cell sources. All G2019S lines evaluated had decreased viability after H_2_O_2_ treatment. The Cj-iPSC clone 1 did not survive to the time point of the viability assay so it was not included in the analysis. It was not clear if this was an artifact or a consequence of ‘aged’ LRRK2 G2019S neurons in culture, since soon after the Cj-ESC clone 16 also could no longer be maintained due to excessive cell death. Interestingly, phenotypes observed in Clone 31 were more robust despite a significant decrease in LRRK2 expression. However, based on our tLRRK2 data, and the literature on using LRRK2 inhibitors^32^, we do not expect decreased functional LRRK2 to present with these phenotypic effects. Therefore, we predict the observed phenotypes in Clone 31 are a result of the increased kinase activity. The evaluation of RNA and protein expression associated with autophagy, ER stress, apoptosis, and the mitochondrial fission protein DRP1 indicated some significant increases of transcripts *P62*, *BIP*, *CHOP* and *LC3* in mutant clones compared to parental wild type controls but there was not a robust marked change in all G2019S clones. Assessment of P62 and LC3 protein levels showed significant decreases of both in Cj-iPSC LRRK2 G2019S neurons compared to wild type, suggesting autophagic dysfunction as well as translational regulation with contrasting RNA and protein changes. Although similar to the transcript analysis, the results were not robust across all G2019S lines. Importantly, the autophagy/lysosome system, unfolded protein response pathways, ER stress, and mitochondrial dysfunction are known to either directly or indirectly be affected by LRRK2 G2019S^33,34^. As aforementioned, we hypothesize that the variability observed across lines is due to the inconsistent abundance of non-neuronal cells. It is possible that the differences in TH+ differentiation also plays a role, however, due to the low (10–20%) TH+ abundance, any discrepancies among lines are likely to be overshadowed by the TH negative neurons or MAP2 negative progenitor populations. This point is critical as undifferentiated cells present a lower abundance of LRRK2 expression compared to differentiated neurons. Future evaluation of additional parental cell lines is warranted to increase the number for the baseline readouts and differentiate between the effect of reprogramming vs. parental cell line variability.

Dopaminergic neurons derived from human iPSC LRRK2 G2019S carriers, typically present reduced neurite complexity^2,5^. Similarly, morphological analysis demonstrated less arborization complexity in 3 of 4 mutated marmoset cell lines. It should be noted that the morphological analysis was the only specific evaluation in this study for TH+/MAP2+ neurons. Interestingly, decrease in neurite and branching parameters only reached significance in one G2019S clone. A difficulty we encountered with this evaluation was the dramatic difference in neuronal morphology dependent on proximity to other neurons or progenitor cells. To optimize reproducibility, the neurons were plated at very low density (75,000 per 35 mm well) to avoid morphological variability simply as a result of more adjacent progenitor cells. Whereas an increased density would make the identification of individual neuron morphology challenging. The lower cell density may have had a ceiling effect on the propensity for complex morphology as the neurons maintain more simplified morphology and, in many cases, bipolar migrating morphology, thus limiting the detection of more significant differences. Strategies that employ isolating mature neurons from progenitor populations followed by morphological discrimination within higher density cultures would be optimal. Alternatively, the development of TH-GFP reporter plasmids transfected at a low efficiency would solve this issue, allowing for high density culture while still being able to discern individual neurons.

The increase in kinase activity found in PD carriers of the G2019S mutation has led to the development and evaluation of kinase inhibitors as neuroprotective strategies (see review^23^). The recent identification of increase kinase activity in sporadic PD cases, has furthered the interest in this approach^35^. Several LRRK2 kinase inhibitors have shown positive results^32,36,37^. However, side effects have been reported which include the identification of vacuolated type-II pneumocytes in the lungs of cynomolgus monkeys^38,39^. A follow-up study uploaded to BioRxiv by Baptista *et al*. suggests the inhibitor-mediated lung pathology is acute and reversible. Another study by Fuji *et al*.^38^ questioned the safety of kinase inhibitors, in particular the potential for downstream impact of the loss of LRRK2 function. In our experiments, the truncation of the LRRK2 protein through CRISPR/Cas9 gene editing did not produce any detectable detrimental results and provides an alternative method for decreasing kinase activity in a more targeted manner. Follow-up experiments using kinase inhibitors applied to both LRRK2 G2019S and WT neurons in parallel to LRRK2 truncated neurons would be an interesting comparison to extrapolate the role LRRK2 plays in these observable cellular phenotypes.

Our results with the CRISPR/Cas9 point mutations in marmoset stem cells, and subsequent phenotype analysis of LRRK2 G2019S cells confirms that the common marmoset is a valid candidate species for a nonhuman primate monogenic model of PD. Critically, the LRRK2 G2019S mutation increased the kinase activity of marmoset LRRK2 as demonstrated by the increase in pS1292 prevalence. These results support our goal of developing a genomic edited LRRK2 G2019S monkey model of PD. Progress in nonhuman primate PD modeling strategies is crucial, as it may lead to the discovery of biomarkers and early intervention strategies aimed at halting or reversing the disease course. This report represents one more step towards having a translational representation of the progressive and systemic pathology that occurs in PD patients.

## Methods

### Stem cell culture and neural differentiation

Cj-ESCs (obtained from the lab of Dr. James Thompson, University of Wisconsin) and iPSCs^17^ were grown in stem cell medium (SCM) consisting of E8 medium (A14666SA, Life Technologies), E8 supplement (A15171-01, Life Technologies), nodal (100 ng/ml, 3218-ND, R&D Systems), Glutamax (35050-061, Life Technologies), chemically defined lipid concentrate (11905-031, Life Technologies), and reduced glutathione (1.94 μg/ml, G4251, Sigma). Neural induction medium composed of DME/F12 (SH30023.01, Thermo Scientific), MEM-NEAA (11140-050, Gibco), N2 Supplement (17502-048, Gibco), SB431542 (10 μM, 04-0010, Stemgent), DMH1 (200 nM, 4126, Tocris), SHH (500 ng/mL, 464-SH, R&D Systems) and CHIR99021 (0.4 μM, 04-0004, Stemgent) was added one day after passaging ~50,000 cells/35 mm well. On day 4, SHH was substituted for purmorphamine (5 μM, 04-0009, Stemgent). By day 8, colonies were lifted from the culture surface with dispase (07923, StemCell Technologies) and grown in suspension as neurospheres for 20 days. After 12 days, SB431542 and DMH1 were removed, purmorphamine was reduced to 0.2 μM and FGF8b (100 ng/mL, 100-25, Peprotech) was added. On day 28, neurospheres were then dissociated, plated to coverslips, and maintained in neural differentiation medium (NDM) consisting of neural basal medium (21103-049, Gibco), MEM-NEAA (11140-050, Gibco), N2 Supplement (17502-048, Gibco), B27 supplement (17504-044, Gibco), brain-derived neurotrophic factor (BDNF; 10 ng/ml, 450-02, Peprotech), glial-derived neurotrophic factor (GDNF; 10 ng/ml, 450-10, Peprotech), TGF-β3 (1 ng/ml, 100-36E, Peprotech), ascorbic acid (200 μM, A0278, Sigma), and cyclic adenosine 3’, 5’-monophosphate (cAMP; 1 μM, A9501, Sigma). Neural differentiation has been previously described in detail, see Vermilyea *et al*.^17^.

### CRISPR/Cas9 genomic editing

Precise editing to obtain the LRRK2 g.G6055A/p.G2019S mutation was achieved using a lentiCRISPR-V1-gRNA/puromycin selection expression cassette (Addgene: #49535). The gRNA variable domain was designed utilizing the g.G6055A site as part of the PAM to prevent subsequent Cas9 binding after homology directed repair (Fig. 1). The vector was introduced through electroporation (BioRad Gene Pulser; 250 V, 500 μF, 4 mm cuvette) in a total volume of 600 μl buffer that included 3.0_E_6 cells, 25 μg plasmid, and 1 nmole of a 141-nucleotide repair template with 70 nucleotide-flanking arms. The cells within an individual cuvette were then immediately plated to one well of a 6-well plate with SCM (supplemented with Y27632, Tocris catalog no: 1254; and L755507, Sigma catalog no: 1362), and 24 hours later 0.5 μg/mL of puromycin was added to the medium for 72 hours. Surviving colonies were isolated, expanded, and evaluated for the homology-directed g.G6055A repair through Sanger (UW-Biotechnology Center Sequencing Core) and next-generation sequencing (NGS; Massachusetts General Hospital (MGH) DNA Core).

The truncated LRRK2 cell line was edited using wild type Cas9 and a gRNA species that guided Cas9 to cut the DNA 23 base pairs in the 3’ direction from the g.G6055 site. The 20-nucleotide variable region was cloned into a vector containing wild type Cas9 and green fluorescent protein (GFP) (Addgene: #48138). The vector was introduced through electroporation (Amaxa nucleofector; Program A-013) using 0.5_E_6 cells and 10 μg expression plasmid in 100 μl of nucleofector buffer solution (VPH-5012, Lonza) and GFP expressing cells were isolated through fluorescent activated cell sorting and evaluated by SURVEYOR Mutation Detection Kit (706020, IDT) as well as Sanger and next-generation sequencing.

All mutant lines were sequenced using NGS (MGH DNA Core) to confirm the homogeneity of the single isolated clones and rule out any potential wild type or mutant contamination of the lines.

### Off-target analysis

The gRNA target sequence for G2019S: ATTGCAAAGATTGCTGACTA and tLRRK2: GCTCAGTACTGCTGTAGAAT with adjacent NGG PAM sites were input into the CRISPR RGEN Tools Cas-OFFinder (http://www.rgenome.net/cas-offinder/) to detect potential off-target binding domains within the Cj-genome. The eight most homologous loci that included an immediately adjacent PAM site were chosen for further Sanger sequencing analysis (see Supplemental Table 3 for primer sequences).

### Immunofluorescence

Immunofluorescent staining was performed to identify markers of neuronal differentiation as well as regional patterning. Coverslip staining was performed as previously described (Vermilyea *et al*.^17^) using the primary antibodies (see Supplemental Table 4) against βIII-Tubulin, microtubule associated protein 2 (MAP2), tyrosine hydroxylase (TH), FOXA2, and PAX6 followed by the secondary antibody AF 488 donkey anti-mouse (A21202, Invitrogen) and Cy3 donkey anti-mouse (715-165-150, Jackson). Coverslips were then counterstained with phalloidin and/or DAPI. Images were taken with a Nikon A1 confocal microscope. For differentiation efficiency analysis, each data point represents a separate captured field.

### Morphological analysis

Dopaminergic differentiated neurons (day 60 of neural induction) were plated at a density of 75,000 cells per 35 mm well and grown for one week before fixation with 4% PFA. Coverslips were then immunostained against MAP2 and TH to identify dopaminergic neurons; n = 25–45 neurons per line. Images were taken with a Nikon A1 confocal microscope. Neurite measurements were obtained using the plugin, “Simple Neurite Tracer,” from ImageJ software. The plugin automatically traced the neurite, after the beginning and endpoint were manually selected, and calculated the following: cell count, neurites per cell, neurite length per cell, branches per neurite, and branches per cell.

### Quantitative-(q) RT-PCR

RNA was isolated from cells (n = 3 technical replicates per line) using a Trizol reagent (15-596-018, Thermo Fisher Scientific) extraction method. cDNA was synthesized using the SuperScript III First-Strand Synthesis System (18080051, Invitrogen) from 0.5 µg of RNA template. qRT-PCR reactions were carried out using iQ SYBR Green Supermix (Bio-Rad, Cat# 1708882). Controls for the qRT-PCR experiment included technical triplicates, no reverse transcriptase controls for each primer set, and normalization to the amplification of the reference gene GAPDH (Glyceraldehyde 3-phosphate dehydrogenase) was used to calculate the dCT for each gene. All primer pairs (see Supplemental Table 3) were screened for gene amplification specificity using the standardized PCR temperature parameters (Initial denature: 95 °C, 10 min; Amplification: 95 °C, 30 s, denature; 55 °C, 30 s, annealing; 72 °C, 30 s, extension; Final extension: 72 °C, 5 min) followed by agarose gel electrophoresis.

### H2DCF-DA reactive oxygen species assay

The assay was administered 48 hrs after seeding 20,000 cells per well of a white opaque 96-well plate (Corning, 3903); n = 3 separate wells per line and condition. Cells were washed three times with DME/F12 prior to treatment with 1 mM H2DCF-DA (ThermoFischer, C400) in DME/F12 for 60 minutes at 37 C. After three washes, DME/F12 or 0.1%, 0.5% or 1.0% hydrogen peroxide was added to the cells and DCF fluorescence was read using a Synergy plate reader (Filter: ex.485/20, em.528/20). Independently, pure DCF compound was serially diluted to establish a log10 based standard curve to extrapolate DCF concentration to fluorescence output (Supplemental Fig. 1). To verify seeding consistency, cells were post-fixed with 4% PFA and stained with DAPI. DAPI fluorescence was then quantified using the Synergy plate reader (Filter: ex.360/40, em.460/40). To interpolate the respective cell number, cells were independently plated using halving dilutions and fixed 3 hours later. A standard curve was established to relate DAPI fluorescence to cell number.

### Transcription factor expression quantification

Differentiated cells were fixed and stained for FOXA2 or PAX6 on coverslips and imaged using a Nikon A1 confocal microscope. For quantification, the images were counted manually or automatically using the ImageJ plug-in “Cell Counter.”

### Viability assay

Differentiated neurons were passaged and plated at a concentration of 40,000 cells per well to a 96-well culture plate. After 48 hours, sterile MilliQ-water, 0.1% H_2_O_2_, 0.5% H_2_O_2_, or 5% DMSO was added to triplicate wells. One hour later, 10 μl of EZQuant solution (EZQuant Cell Quantifying Kit; Alstem; Richmond, CA) was added to each well and incubated at 37 °C. Absorbance was read at 450 nm using a microplate reader 3 hours after the addition of the EZQuant solution.

### Lysate preparation

100 µl of 1X Cell Signaling lysis buffer (#9803), with protease and phosphatase inhibitors (#78430 and #78427, respectively), was added to dopaminergic cell pellets and samples were left on ice to lyse for 30 minutes, n = 3–4 separately differentiated and collected samples per line. The lysates were then transferred to a 1 mL Eppendorf tube and centrifuged at ×20,000 g for 10 minutes at 4 degrees Celsius to pellet cell debris. The Pierce™ Bovine Serum Albumin Standard Pre-Diluted Set (#23208) was used to obtain protein concentrations of each supernatant.

### Western blots

Samples were supplemented with NuPage LDS sample buffer 4× (NP0008), boiled for 5 min at 95 °C and 50 µg of protein per sample were run on a Bio-Rad Criterion™ TGX™ polyacrylamide gel (Cat #5671095) at 200 V for 37 min. Gels were transferred to nitrocellulose (Bio-Rad, Cat #1704159) on a Bio-Rad Trans-Blot® Turbo™ transfer system at 20 V for 10 min. The nitrocellulose was blocked for 1 h in 50% TBS (20 mM Tris, 0.5 M NaCl, pH 7.5), 50% Odyssey blocking buffer (Li-Cor; 927–40,000) and incubated overnight with primary antibodies in 50% TBS-T (20 mM Tris, 0.5 M NaCl, pH 7.5, 0.1% Tween 20) and 50% Odyssey blocking buffer at 4 °C. Following 3 × 5 min washes with TBS-T, the nitrocellulose was incubated at RT with IR Dye 800CW Goat anti-rabbit IgG (#926-32211) or IR Dye 680RD Goat anti-mouse IgG (#926-68070) secondary antibodies for 1 h, washed 3 × 5 min and scanned on the Li-Cor platform. A list of antibodies can be found in Supplemental Table 4 and uncropped blots in Supplemental Fig. 5.

### Statistical analysis

Prism 8 (GraphPad Software, Inc., 2018) was used for all statistical analyses. Where applicable, normality was tested using either D’Agostino-Pearson omnibus or Shapiro-Wilk tests prior to choosing the appropriate statistical test. One-way ANOVA or Kruskal-Wallis test with Tukey’s or Dunn’s correction for multiple comparisons, two-way ANOVA, and Student’s t-tests were performed where appropriate. Specific statistical analyses are described in the legends of the respective figures.

## Supplementary information

Supplemental Figures and Tables.
