# Supplementary material for: In Vitro CRISPR/Cas9-Directed Gene Editing to Model LRRK2 G2019S Parkinson’s Disease in Common Marmosets
Source: Sci Rep. 2020 Feb 26;10:3447. doi: 10.1038/s41598-020-60273-2 (PMC7044232; doi:10.1038/s41598-020-60273-2)
Supplement: Supplementary file 1 — Supplemental Figures and Tables. [file 41598_2020_60273_MOESM1_ESM.pdf]

SUPPLEMENTAL FIGURES AND TABLES

In Vitro CRISPR/Cas9-Directed Gene Editing to Model LRRK2 G2019S Parkinson's Disease in Common Marmosets

Scott C. Vermilyea, Alexander Babinski, Nina Tran, Samantha To, Scott Guthrie, Jillian H. Kluss, Jenna Kropp Schmidt, Gregory J. Wiepz, Michael G. Meyer, Megan E. Murphy, Mark R. Cookson, Marina E. Emborg, Thaddeus G. Golos

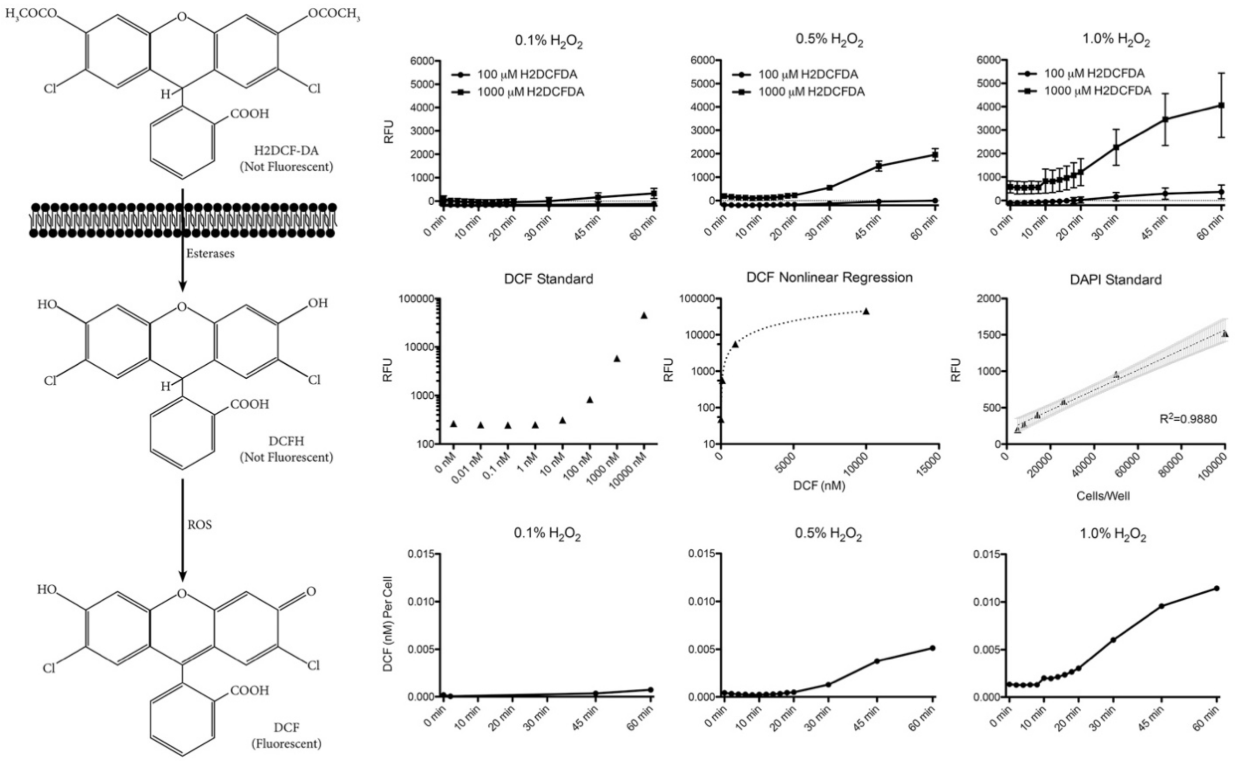

Supplemental Figure 1: H2-DCFDA Optimization and Interpolation. H2DCF-DA is a cell permeable compound that converts to a fluorescent reporter when exposed to ROS. H2DCFDA concentration and plating density variability was optimized for the neuronal culture. RFU was controlled through a DCF standard nonlinear regression as well as a DAPI standard linear regression to interpolate the concentration of DCF per cell.

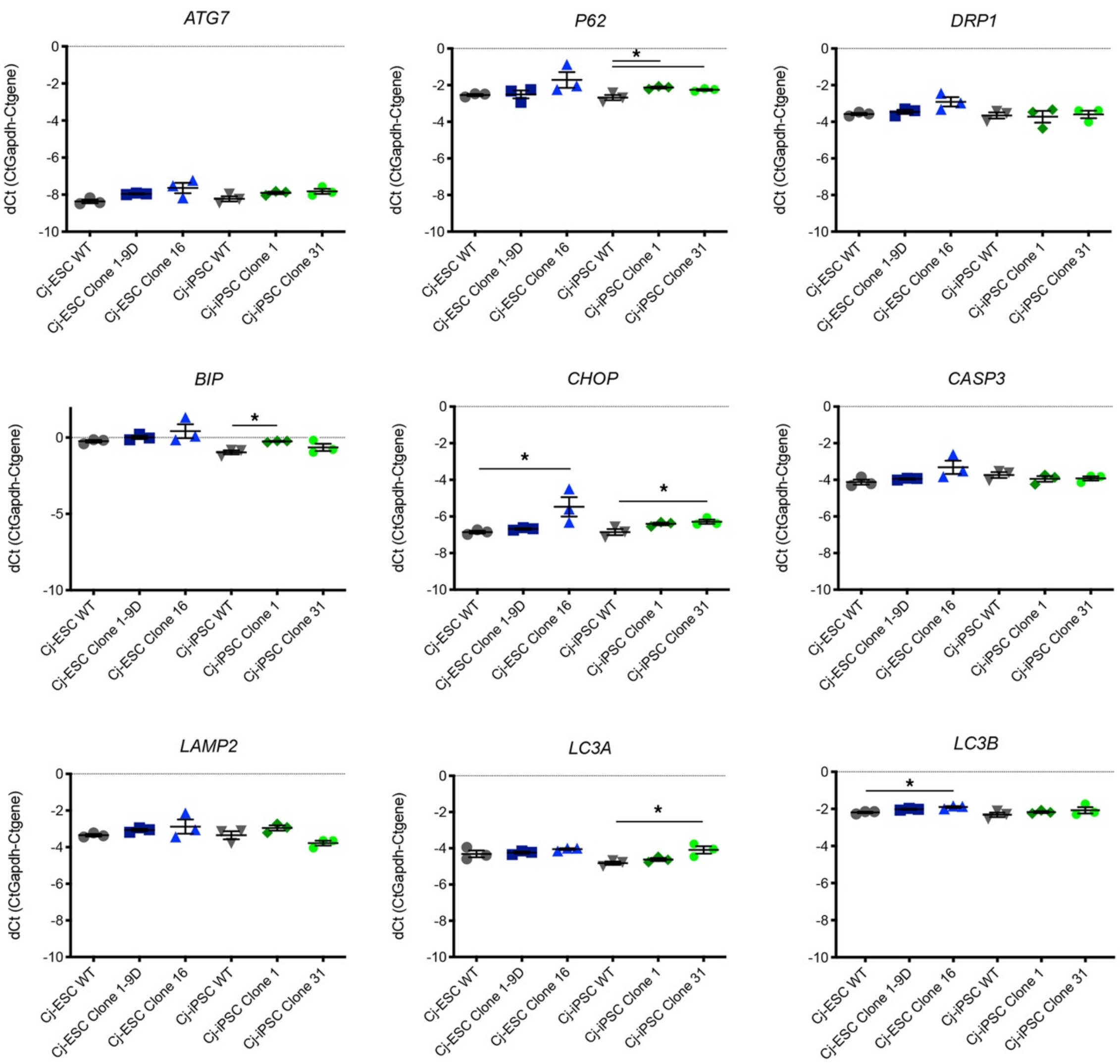

Supplemental Figure 2: qRT-PCR for autophagy, ER stress, apoptotic, and mitochondrial related mRNAs. Expression of *ATG7*, *P62*, *DRP1*, *BiP*, *CHOP*, *CASP3*, *LAMP2*, *LC3A*, and *LC3B* from samples collected on d67 of dopaminergic differentiation. (One-way ANOVA with Tukey's multiple comparison was used to compare among Cj-ESC or Cj-iPSC lines; $p < 0.05^{*}$)

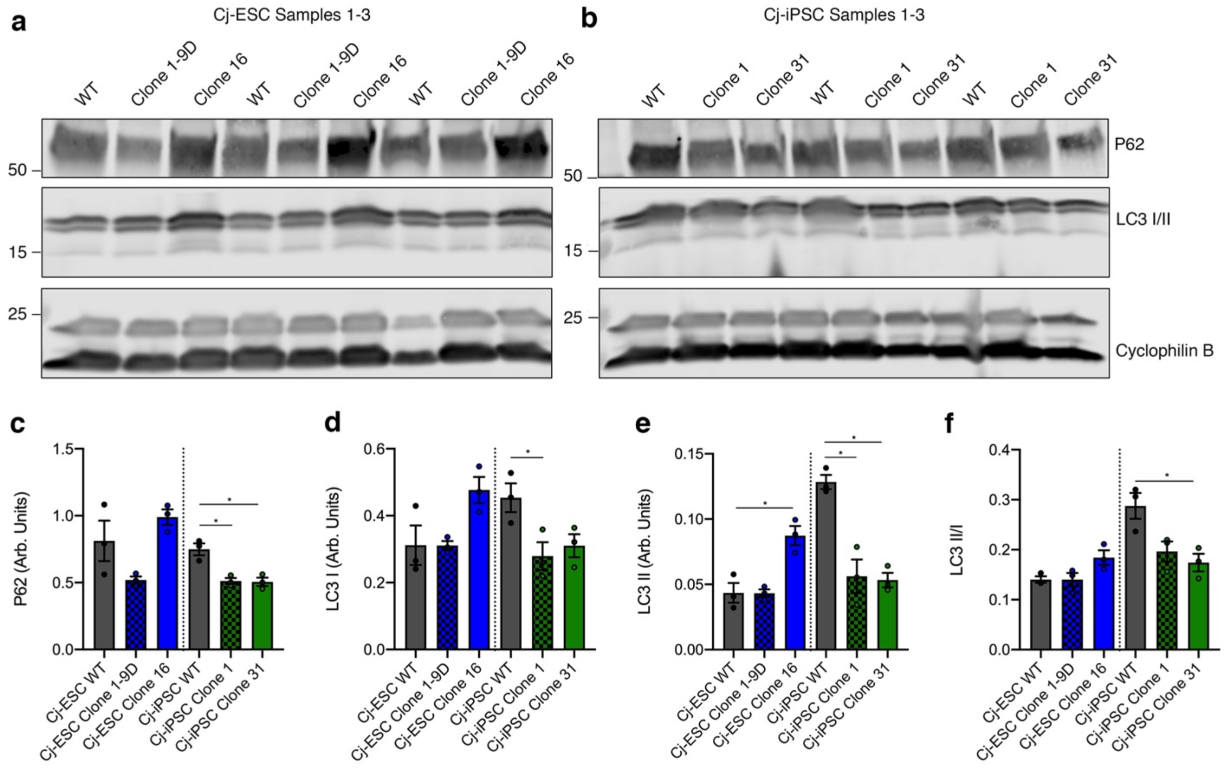

Supplemental Figure 3: Western blot for autophagy-related proteins. (**a & b**) Blot for P62, LC3I-II, with Cyclophilin B as the loading control. (**c**) Quantification of P62, (**d**) LC3I, (**e**) LC3II, and (**f**) LC3II/LC3I for Cj-ESC and Cj-iPSCs. (One-way ANOVA with Tukey's multiple comparison was used to compare among Cj-ESC or Cj-iPSC lines; $p < 0.05^{*}$; Arbitrary units (Arb. units)

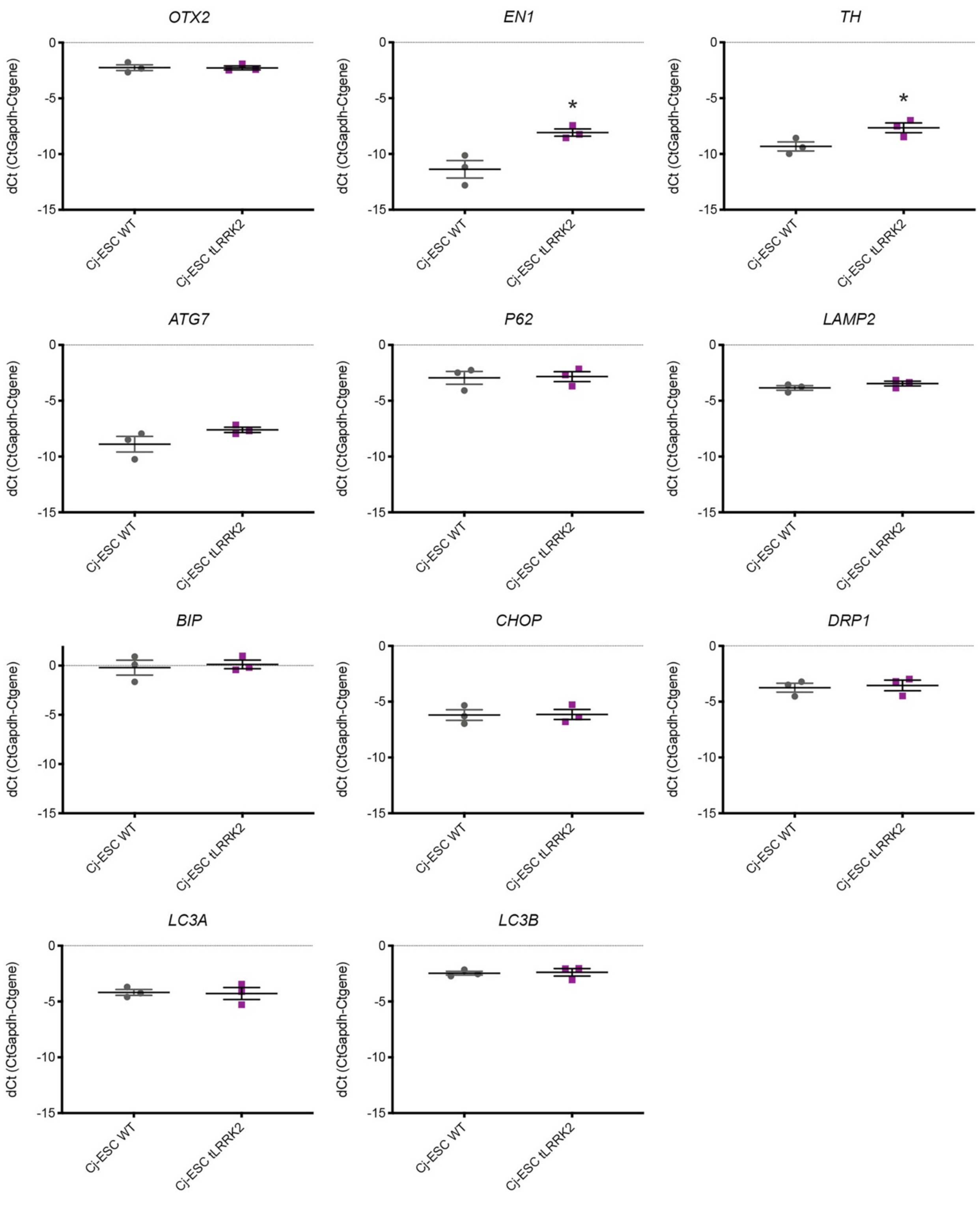

Supplemental Figure 4: Evaluation of midbrain dopaminergic identity and autophagy/ER stress related genes. Gene expression by qRT-PCR to evaluate dopaminergic patterning and differentiation, as well as autophagy and ER stress related genes. Student t-tests were used for statistical analysis.

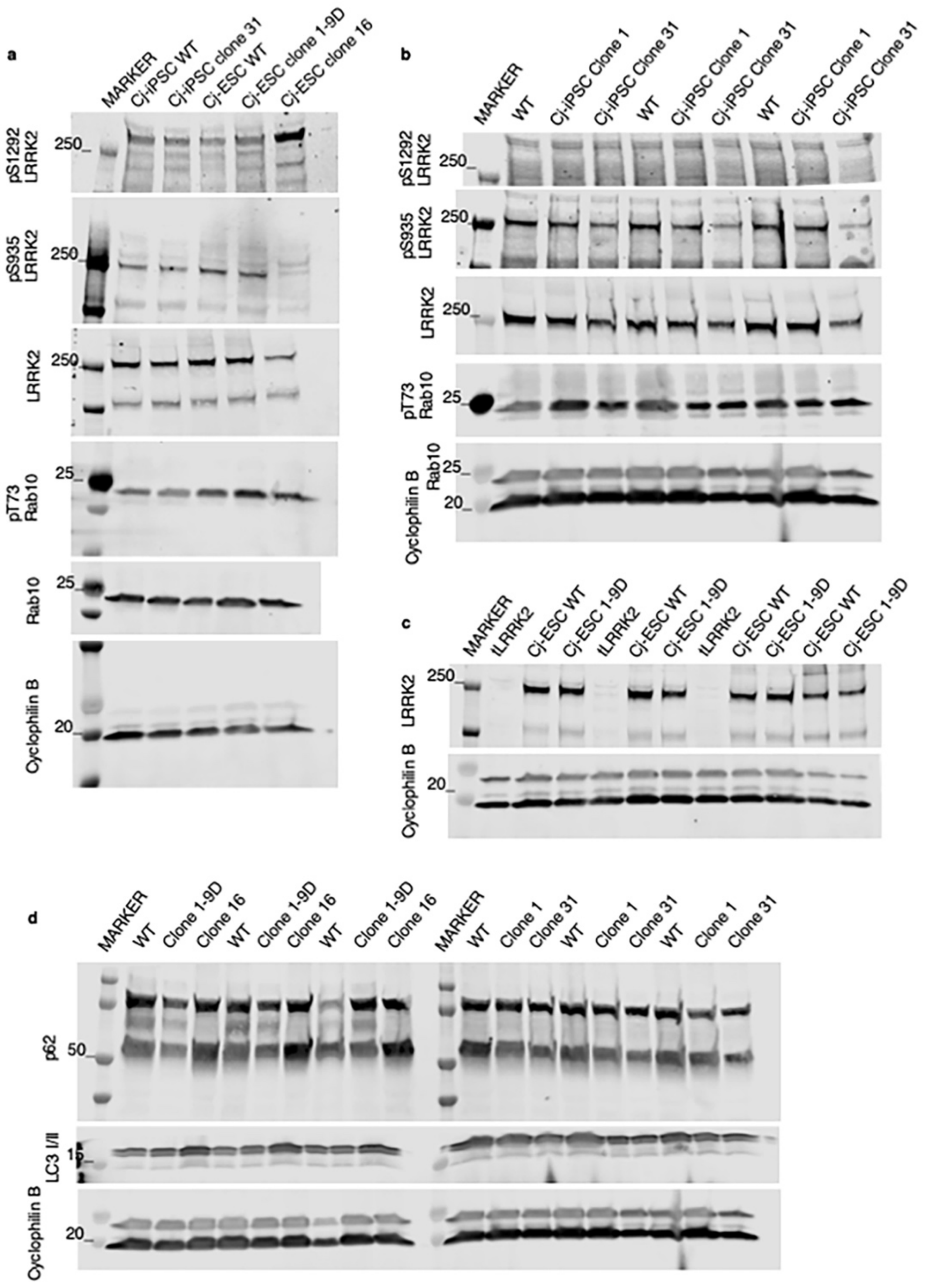

Supplemental Figure 5: Uncropped blots of figures 3, 6 and supplementary figure 3 with molecular weight markers. (**a,b**) Uncropped blots of figure 3a,b; Western Blots for pS1292 LRRK2 autophosphorylation, pS935 LRRK2, LRRK2, pT73 Rab10, Rab10, and cyclophilin B for Cj-ESC wild type (WT), Cj-ESC Clone 1-9D, and Cj-ESC Clone 16, and (**b**) Cj-iPSC WT , Cj-iPSC Clone 1, and Cj-iPSC Clone 31. (**c**) Uncropped blot of figure 6f; Western blot for LRRK2 using a C-terminal antibody shows the loss of LRRK2 detection in Cj-ESC tLRRK2 neurons. (**d**) Uncropped blot of supplementary figure 3a,b; Western blot for P62, LC3I-II, with Cyclophilin B as the loading control.

			Off-target mutation			
Potential off-target sites	Gene	Input sequence: ATTGCAAAGATTGCTGACTANGG	Cj-ESC Clone 1-9	Cj-ESC Clone 16	Cj-iPSC Clone 1	Cj-iPSC Clone 31
Chr 1: 111645639-111645661	Unknown	ATTGCTAAGATTGCTGCTTATGG	No	No	No	No
Chr1: 162034774-162034796	Unknown	ATTGGAAATATTGCTGACTCAGG	No	No	No	No
Chr2: 112903883-112903905	Unknown	ATTACCAAGCTTGCTGACTAAGG	No	No	No	No
Chr2: 113693146-113693168	Unknown*	ATTGCAAAGTTTTCTGAATATGG	No	No	No	No
Chr2: 4024878-4024900	Unknown	ATTTGAAAGATTGCTGCCTAGGG	No	No	No	No
Chr2: 85087588-85087610	Unknown	ATTGCAATGAGTGCTGACAAAGG	No	No	No	No
Chr3: 121493937-121493959	Unknown	ATTGCCAAGATTGTGGACTAAGG	No	No	No	No
Chr5: 71505305-71505327	Unknown	ATTTCAAGGATTTCTGACTAAGG	No	No	No	No

Supplemental Table 1: CRISPR gRNA potential off-target sequence analysis. Two separate g.G6055A clones from Cj-ESCs and Cj-iPSCs were isolated and homogeneity was verified through next-generation sequencing. The top eight predicted off-target sites were evaluated for INDEL formation. None of the sequences are within annotated genes in the marmoset or human, except for one region on human chromosome 2 which was predicted as part of the transcript variant for METTL22 and SLC25A53 in the human (indicated with *).

			Off-target mutation
Potential off-target site	Gene	Input sequence GCTCAGTACTGCTGTAGAATNGG	Cj-ESC tLRRK2
Chr2: 143675482-143675460	Unknown	GCTAAATTCTGCTGTAGAATTGG	No
Chr9: 84780276-84780298	Unknown	GCTCAGTACTGCTGTAATACAGG	No
Chr10: 45159485-45159463	Unknown	GCTCAGCAATGCTGTAGATTAGG	No
Chr11: 122658242-122658220	Unknown	GCTAAGTTCTGCTGTAGAATAGG	No
Chr15: 39184321-39184343	Unknown	CCTCTGTACTGCTGTAGAAGAGG	No
Chr16: 61184568 61184546	Unknown	GCTCAGTACTGCTGGAGGCTGGG	No
Chr20: 4367998 4368020	Unknown	GCTCAGCACTGCTGTAAAATGGG	No
Chr22: 49084681 49084659	Unknown	GCTCAGTACTGCTGCAGCACCGG	No

Supplemental Table 2: CRISPR tLRRK2 gRNA off-target sequence analysis. The top eight potential off-target sites were evaluated for INDEL formation.

Supplemental Table 3: qRT-PCR and DNA sequencing primer pairs

Differentiation		
**Gene**	**Pair**	**5'-Sequence-3'**
NANOG	Fwd	GATGTGACTCAGAAGGCCTCA
	Rev	CCATACCGGAAGGTTCCCAG
NEUROD1	Fwd	CTGTCACCGCTCAGGACCTA
	Rev	GTCCAGCTTGGAGGACCTTG
OTX2	Fwd	CGAGAAGGATGTGGTCCGTG
	Rev	ACACTGGTCCCCCTGAGAAA
EN1	Fwd	GATCGTCCATCCTCCGGTCC
	Rev	CGATAGCACCTGTCCGAGTC
TH	Fwd	CTTGCACTCCCTGTCTGAGG
	Rev	TGTCTGGTCTTGGTAGGGCT
Autophagy-related Gene		
**Gene**	**Pair**	**5'-Sequence-3'**
ATG7	Fwd	CCGGAAGTTGAGTGTTGGATT
	Rev	TCCTCTGTAGTTGCTGCCAT
P62	Fwd	ACCCTGAGGAACAGATGGAATC
	Rev	TCCAAAGAGCTTGGCCCTTC
LAMP2	Fwd	TCATGTTGTGCTTCCGCCTC
	Rev	TAAGACCGCAAGGCTCCCAG
LC3A	Fwd	CGTAGTCAGAGGGCACCAAC
	Rev	GATGAACCACACAGCAGGAGT
LC3B	Fwd	GCACCTTCGAACAAAGAGTAGA
	Rev	GCTTCTCACCCTTGTATCGTTC
ER/Apoptosis/Mitochondria & Reference		
**Gene**	**Pair**	**5'-Sequence-3'**
BIP	Fwd	TCCCGAGAACACGGTCTTTG
	Rev	TTCAACCACCTTGAACGGCA
CHOP	Fwd	CGATCCCTAGGTCAGAGGCT
	Rev	CCTCCTTGAACACTCTCTCCTC
CASP3	Fwd	ACCAATGGACCTGTTGACCTG
	Rev	CCACGGCAGGCCTGAATAAT
DRP1	Fwd	CAGCAATGGAGGCGCTAATTC
	Rev	TCTTTCCACTGCTCTGCGTTC
GAPDH	Fwd	TCGGAGTCAACGGATTTGGT
	Rev	ATGGCGACGACATCCACTTT
LRRK2 g.6055 Region Sequencing Primers		
**Method**	**Pair**	**5'-Sequence-3'**
Sanger	Fwd	GGGTCAAAGTGAGCACAG
	Rev	GTGGACCTGAATTTGACTCACC
NGS	Fwd	CCTGTGGAATTTCTGGCAG
	Rev	CTTCTGACTCTTCTGAACTC

LRRK2 G2019S Off-Target		
**Chromosome**	**Pair**	**5'-Sequence-3'**
Chr1-1	Fwd	GTAACAAGGCACCAACCACAAG
	Rev	CCAGGCTCTCAGCTAAGCATTC
Chr1-2	Fwd	GGTGCTTGTATGACCTGGTGC
	Rev	TCAAAGAGCAAGTACCCGAGAC
Chr2-1	Fwd	ATGCAAGGCTCCTACTTTCTGG
	Rev	CTTTGGTTGCCAAGATGACAGG
Chr2-2	Fwd	TGAATCCTGGCAAGGCAAGTG
	Rev	CAAGAATTAGCCAGGCATGGTG
Chr2-3	Fwd	TCTTCTTTCCACTTCCCTGGTC
	Rev	GCAAGAAACCATTCCCACAGAG
Chr2-4	Fwd	TGCTAGGTAGGCTTTGACATGG
	Rev	GGCAACTGCTGGCTAATGAAG
Chr3	Fwd	ATGACCAAGGGCTGTGTGC
	Rev	TGGCAACAGAGCAAGACTCC
Chr5	Fwd	GACCCTGCCCTGGACAAATG
	Rev	TGAGGAGTTAGGCCACCAGG
Chr10	Fwd	TCCGAGGGACTCAAACAGC
	Rev	GCTCTGGTTCTTCCTGCTGTC
tLRRK2 Off-Target		
**Chromosome**	**Pair**	**5'-Sequence-3'**
Chr2	Fwd	AGCATAGCACATGGACAGTCTC
	Rev	TTGCATTTCCCTGACTGGTGAG
Chr9	Fwd	AGGCTGAGGCAAGAGAATCG
	Rev	CCTTCTCTGCTGTGTCCTGTG
Chr10	Fwd	AGGGTGACTCTCAGGAAACTGG
	Rev	TCCTTTGAGTTAAGCTGGCTGC
Chr11	Fwd	AGCTAAGGAAGAAGGAAGCAGG
	Rev	TTCTCGACCAGACGGTAGGTG
Chr15	Fwd	AAGAGGTGTTCTCCCACAGC
	Rev	GGTACAGTTTGGGCCATTGC
Chr16	Fwd	AGCCTCAGCTCCTTACTTGC
	Rev	AAATGCACCAAGCAGCCAC
Chr20	Fwd	TGCTTGTCTCTTGTCCCTGTC
	Rev	TCAGACTGGCCTTGAACTCC
Chr22	Fwd	GGTTTCTCGCCCGTGTGTAC
	Rev	ACCATTCGTGTGCAGTGAGTG

Supplemental Table 4: List of primary antibodies

Antibody	Species	Company	Dilution	Application
pS1292 LRRK2 (MJF-19-7-8)	Rabbit; monoclonal	Abcam (ab203181)	1:2000	WB
LRRK2 (MJFF2)	Rabbit; monoclonal	Abcam (ab133474)	1:2000	WB
pS935 LRRK2 [UDD2 10(12)]	Rabbit; monoclonal	Abcam (ab133450)	1:2000	WB
Cyclophilin B	Rabbit; polyclonal	Abcam (ab16045)	1:2000	WB
pT73 Rab10 (MJF-R21)	Rabbit; monoclonal	Abcam (ab230261)	1:2000	WB
Rab10 (MJF-R23)	Rabbit; monoclonal	Abcam (ab237703)	1:2000	WB
LC3B	Rabbit; polyclonal	Novus Biologicals (NB100-2220)	1:2000	WB
SQSTM1/ p62	Mouse; monoclonal	Abcam (ab56416)	1:2000	WB
βIII-Tubulin	Mouse; monoclonal	R&D (MAB1195	0.5 μg/mL	ICC
MAP2	Rabbit; polyclonal	Millipore (AB5622)	1:500	ICC
TH	Mouse; monoclonal	Immunostar (22941	1:500	ICC
FOXA2/HNF-3β	Mouse; monoclonal	Santa Cruz (sc-374376	0.5 μg/mL	ICC
PAX6	Mouse; monoclonal	DSHB (PAX6)	15 μl/mL	ICC
